# Android Platform Based Smartphones for a Logistical Remote Association Repair Framework

**DOI:** 10.3390/s140711278

**Published:** 2014-06-25

**Authors:** Shao-Fan Lien, Chun-Chieh Wang, Juhng-Perng Su, Hong-Ming Chen, Chein-Hsing Wu

**Affiliations:** 1 Integrated Logistic Support Centre, Chung-Shan Institute of Science and Technology, No.481, Sec. chia an, Zhongzheng Rd., Longtan Shiang, Taoyuan County 325, Taiwan; E-Mails: g9510801@gmail.com (S.-F.L.); boriswu0824@hotmail.com (C.-H.W.); 2 Electronic Engineering Department, Chienkuo Technology University, No.1, Chieh Shou N. Road, Changhua City 500, Taiwan; E-Mails: jasonccw@ctu.edu.tw (C.-C.W.); steven@ctu.edu.tw (H.-M.C.); 3 Department of Electrical Engineering, National Dong Hwa University, No. 1, Sec. 2, Da Hsueh Rd., Shoufeng, Hualien County 97401, Taiwan

**Keywords:** logistics support planning, Logistical Remote Association Repair Framework (LRARF), QR-code, Linear Projective Transform (LPT), ANFIS-based data mining, Android-based platform

## Abstract

The maintenance of large-scale systems is an important issue for logistics support planning. In this paper, we developed a Logistical Remote Association Repair Framework (LRARF) to aid repairmen in keeping the system available. LRARF includes four subsystems: smart mobile phones, a Database Management System (DBMS), a Maintenance Support Center (MSC) and wireless networks. The repairman uses smart mobile phones to capture QR-codes and the images of faulty circuit boards. The captured QR-codes and images are transmitted to the DBMS so the invalid modules can be recognized via the proposed algorithm. In this paper, the Linear Projective Transform (LPT) is employed for fast QR-code calibration. Moreover, the ANFIS-based data mining system is used for module identification and searching automatically for the maintenance manual corresponding to the invalid modules. The inputs of the ANFIS-based data mining system are the QR-codes and image features; the output is the module ID. DBMS also transmits the maintenance manual back to the maintenance staff. If modules are not recognizable, the repairmen and center engineers can obtain the relevant information about the invalid modules through live video. The experimental results validate the applicability of the Android-based platform in the recognition of invalid modules. In addition, the live video can also be recorded synchronously on the MSC for later use.

## 1. Introduction

Identifying circuit modules remotely through the network and replacing invalid ones in time is a crucial issue in logistics. To maintain a high-level of logistics support, large-scale network systems, such as rapid transit systems, telecommunication networks or power systems, should be properly maintained. Specifically, some sub-systems or modules are often very far away from the repair station and the system maintenance is usually very costly. In view of this, the development of remote association repair technology (RART) is in high demand for logistics to reduce repair costs and time. The concept of remote maintenance has been developed for decades, however, in the early period remote maintenance technology was centered on internet technology. In recent years, the applications of remote maintenance technology have drawn much attention with the development of wireless transmission technology. Specifically, in manufacturing [1], control and robot systems [2–4], weapon systems [5] and logistics applications [6], remote maintenance technology plays an important role. Nevertheless, most research seems to focus on the diagnosis of the equipment, but it is generally not an easy task to automatically perform fault detection of invalid modules.

**Figure 1.**
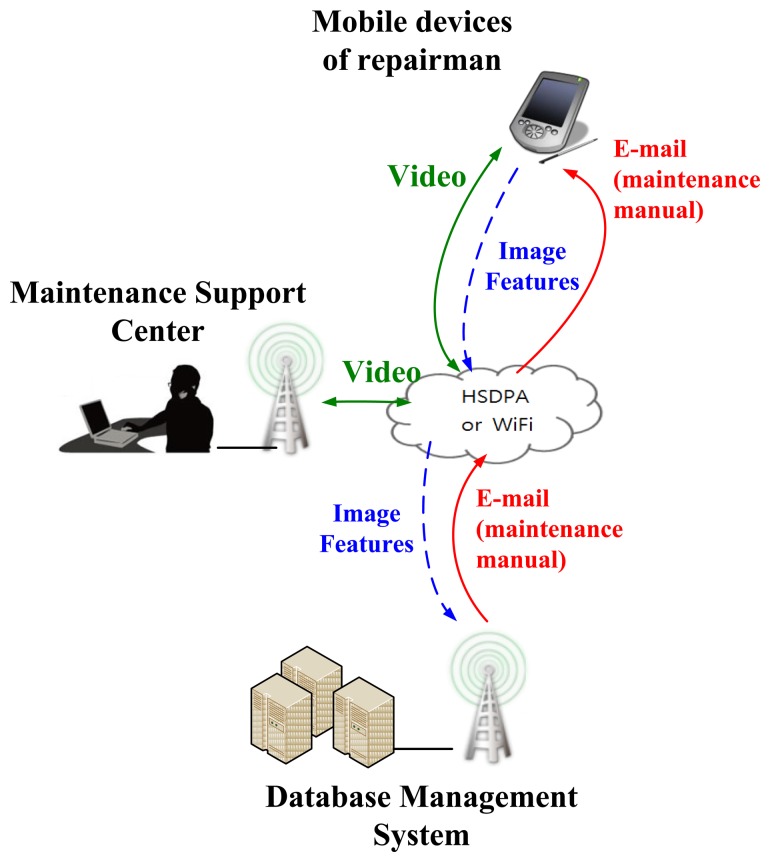
Logistical Remote Association Repair Framework.

In this paper, we have developed a Logistical Remote Association Repair Framework (LRARF) to aid repairmen in efficiently and effectively maintaining the operation of systems. LRARF, as shown in Figure 1, was established for aiding in faulty module detection and maintenance by the integration of QR-code technology, image identification, wireless transmission and intelligent data mining technologies. The architecture of LRARF includes four parts: smart mobile phones, DBMS, a MSC and wireless networks. The transmission of LRARF is performed through High-Speed Downlink Packet Access (HSDPA) or WiFi networks. In this framework, invalid modules' images can be sent back to the DBMS and the MSC through the APP. The experimental results reveal that the images of invalid modules can be sent back to the DBMS and the MSC through the APP and the image recognition algorithm is capable of identifying the invalid module. The corresponding maintenance manual for an invalid module is then sent via e-mail to a smart phone by maintenance personnel. In addition, voice and the live video can be recorded synchronously on the MSC for later use.

## 2. Logistical Remote Association Repair Framework

### 2.1. The Remote Association Repair Framework Process

**Figure 2.**
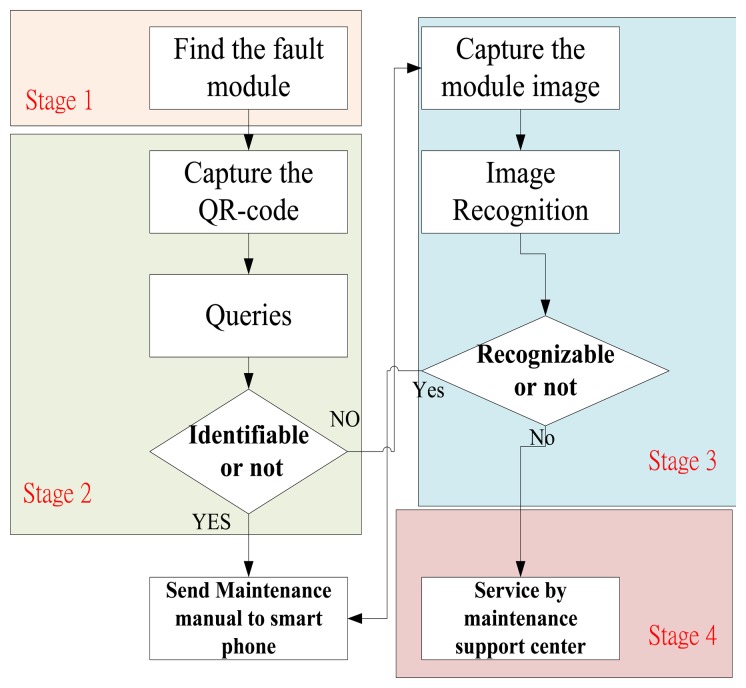
The LRARF process.

The Logistical Remote Association Repair Framework process is shown in Figure 2. In this process, we design four operation stages to identify a module:

*Stage 1* is the invalid module identification by repairmen.
*Stage 2* is the QR-code recognition of invalid modules.
*Stage 3* is the image recognition of invalid modules.
*Stage 4* is to assist identification by the MSC engineers.

In Stage 1, repairmen identify the failed module. If the modules can be identified, then the modules will be repaired. If the modules cannot be recognized, then the repairman may capture the module QR-codes (Stage 2). If the QR-codes are damaged, the repairman uses a smart mobile phone to capture an image of the module (Stage 3). The captured QR-codes and images are transmitted to the DBMS so the invalid modules can be recognized via our algorithm.

Furthermore, the image features will be extracted by our APP and transferred to a cloud database to search for the best matching module. Moreover, DBMS will automatically search for the maintenance manual corresponding to the invalid module and transmit back to the maintenance staff. If modules are not recognizable, the repairmen and center engineers can obtain the relevant information about the invalid modules through live video. Table 1 shows the four-stage operations and situational modes.

### 2.2. Database Management System (DBMS)

**Figure 3.**
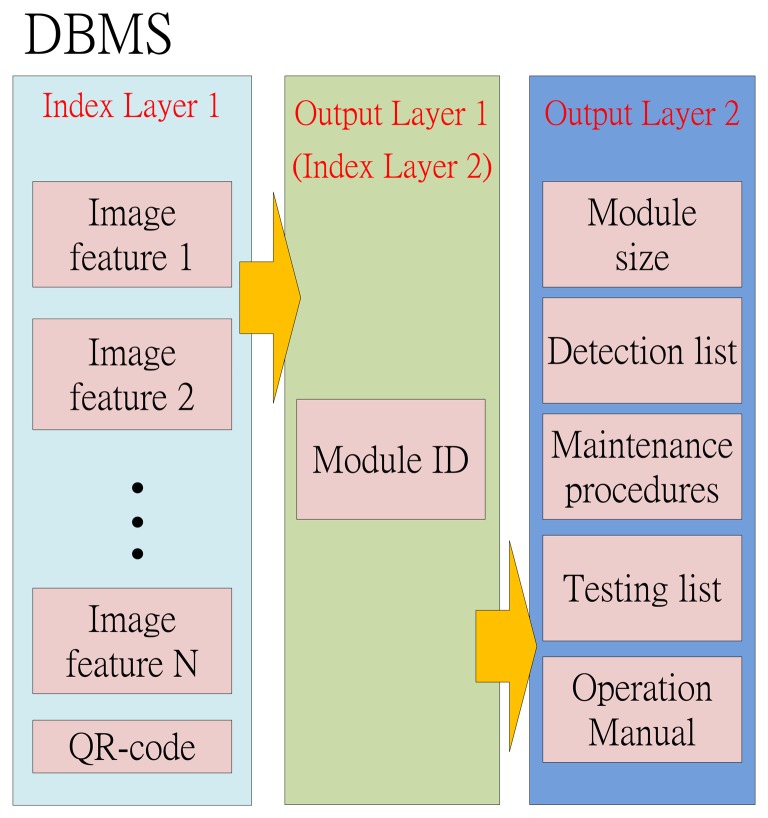
The DBMS architecture.

The architecture of the DBMS is shown in Figure 3. There are three layers in the DBMS. The first layer is called “Index Layer 1”. The image features and QR-codes are stored in this layer. This information will be the indexes for searching the module ID in the second layer of the DBMS. This layer is not only the output layer 1 for the index layer 1, but also the index layer 2 for the next layer. Moreover, the module ID will be the indexes for searching the module sizes, the detection list, the maintenance procedures, the testing list and the operation manual.

## 3. Image Recognition

### 3.1. Android Operating System

**Figure 4.**
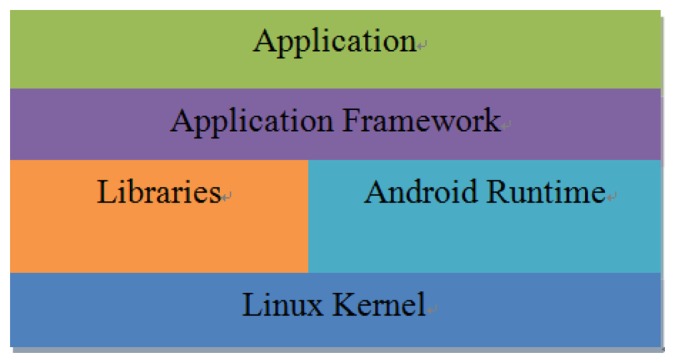
The architecture of Android systems.

**Figure 5.**
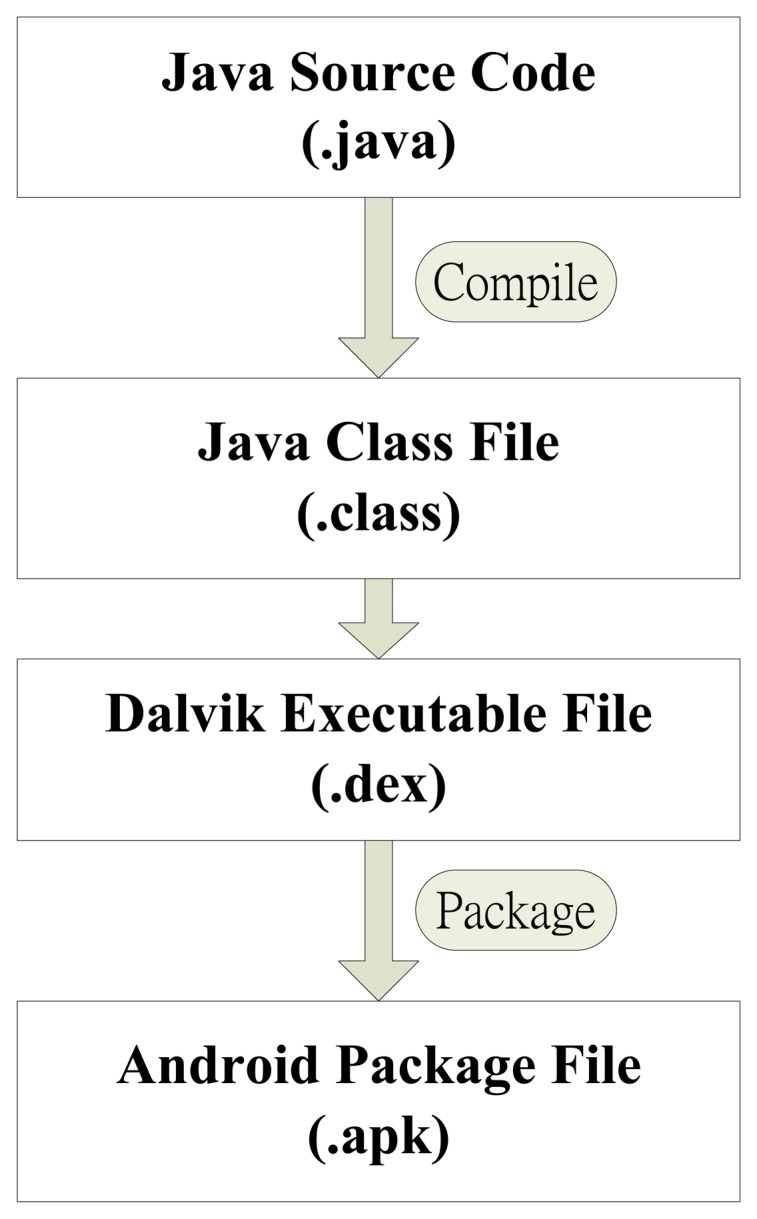
The application program developing process.

Android is an open source operating system (OS) and was released by Google in 2007. The operation structure of Android systems can be divided into five layers: Application, Application Framework, Libraries, Android Runtime and Linux Kernel, as shown in Figure 4. An important feature of the Android OS is that the Google provides free SDK and source code for program developers [7–9]. In this study, Android 2.1 is employed for the application. We developed the video transmission control interface using the Android SDK. Android Runtime is based on the Dalvik Virtual Machine (VM) and Java SE class library. Dalvik VM can be compiled via a Java application. The application program development process is shown in Figure 5.

**Figure 6.**
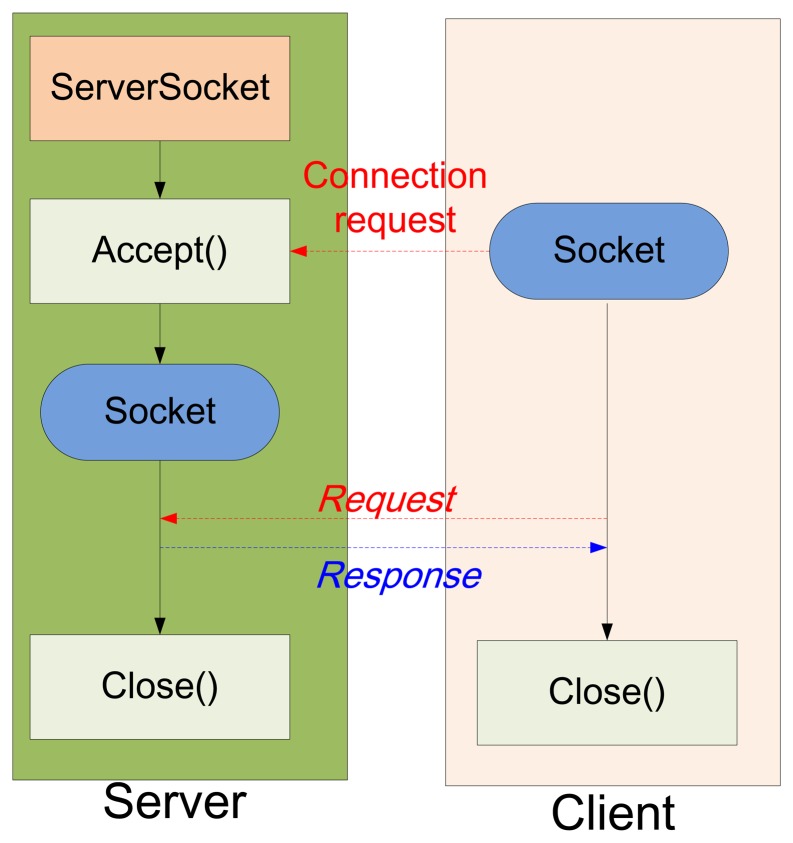
Connection between server and client.

The Android connection between server and client is illustrated in Figure 6. The connection process consists of the following steps [10]:

*Step 1*: initialize the JavaScript function, “ServerSocket()”.
*Step 2*: open the COM port and the JavaScript function is set to “accept()” for dealing with the connection request from the client.
*Step 3*: the server establishes a connection at the request of the client until both sockets of the server and the client are closed.

### 3.2. Image Acquisition Application

**Figure 7.**
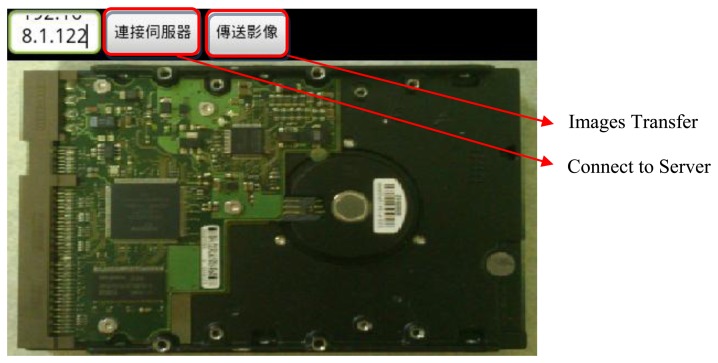
The real-time image capture interface.

**Figure 8.**
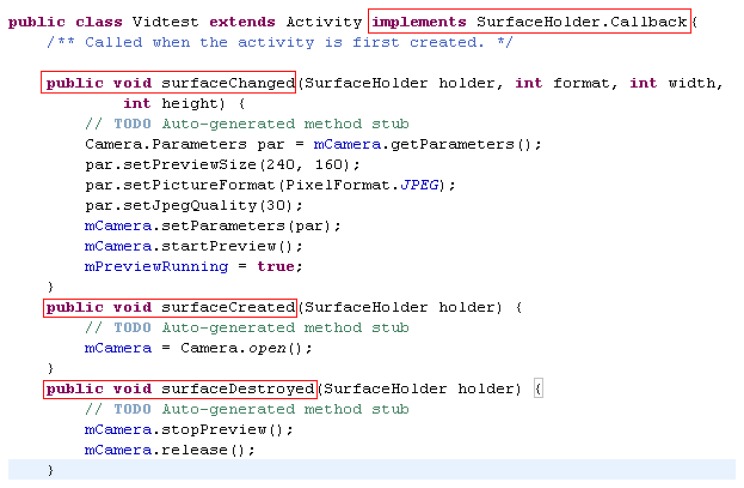
Image acquisition and camera control setting.

An Android-based smartphone is utilized in this research to implement the image acquisition. Android is a Linux-Kernel operation system, which is running on Android Runtime. The program development platform is the free Eclipse Software Development Kit (SDK). Therefore, we choose an Android operated mobile phone to catch images and transmit image data via HSDPA. In this study, we used a Motorola Defy MB525 smart phone as the client and the remote computer as the server. The CPU is a 800 MHz TI OMAP3610 and the development platform is Eclipse version 3.6.2. The dpi of the camera is 5 megapixels and the Java Development Kit (JDK) is JDK 6. Figure 7 shows the real-time image capture interface and Figure 8 depicts the program of image acquisition and camera control settings.

**Figure 9.**
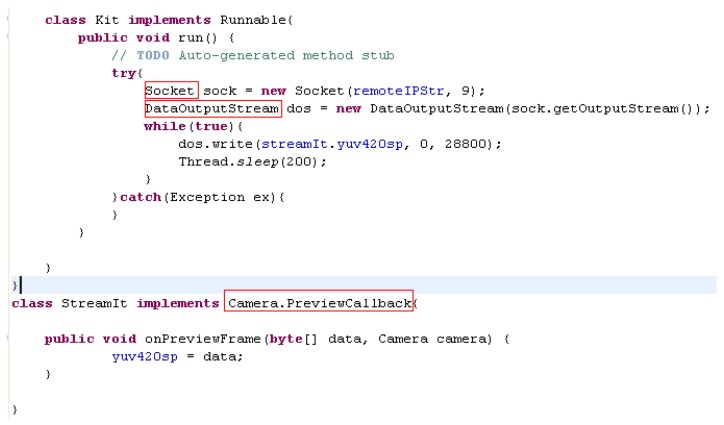
The remote real-time image transformation setting.

**Figure 10.**
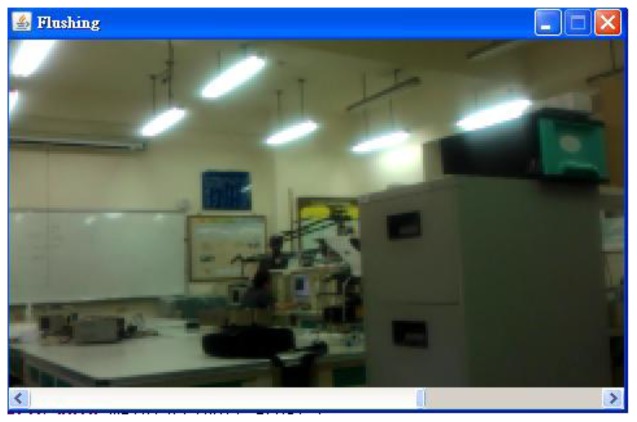
The real-time image on the server computer.

The remote real-time image transformation settings are shown in Figure 9. The function “Camera.PreviewCallback” is exploited for real-time image acquisition and transported to server by the function “DataOutputStream()”. On the other hand, the functions “ColorModel()” and “Raster()” are used for receiving and decoding images on server computer. The result of a real-time image on the server computer is shown in Figure 10, and the remote real-time image transformations are established.

### 3.3. QR-code Recognition

**Figure 11.**
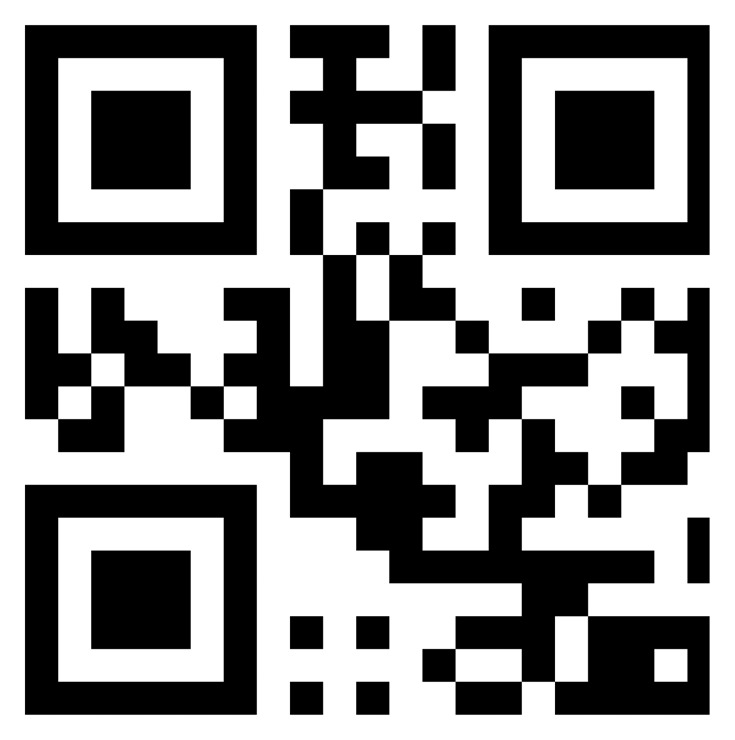
Example of a QR-code.

Barcodes were developed in 1950 and have been widely used in a variety of applications due to their high reliability, efficiency and cost-effectiveness. However, barcodes can only record limited information about items. The 2D-barcode was devised to be capable of including a brief description in addition to assigning a number to an item. Moreover, sound, pictures and even traditional Chinese characters, *etc.*, can be recorded as 2D-barcodes. There are many forms of 2D-barcodes in accordance with various commercial applications. For example, in 1994, the Japan Densoe company [11] developed a rapid reaction barcode (Quick Response code, QR-code) for the array code, shown in Figure 11.

The QR-code design is very clever. The most important features of aQR-code are the position marks of the top left, bottom left corner and the upper right corner. Positioning marks are triple concentric square marks, called a position detection pattern (PDP). Three PDP styles are the 3 × 3 black block, 5 × 5 white block and 3 × 3 black block, respectively. Moreover, the width ratio is 1:1:3:1:1. The patterns are almost impossible to repeat. Therefore, QR-codes can be read quickly by rapid positioning and orientation of the PDP.

The calibration of the QR-code is the crucial technology for QR-code decoding. In this paper, the Linear Projection Transformation (LPT) [12] is utilized for QR-code calibration. There exists a projective matrix between warping QR-code and correct QR-code. The transformation is given by:

(1) $$\left(\begin{array}{c} x_{1}^{\prime }\\ x_{2}^{\prime }\\ x_{3}^{\prime } \end{array}\right)=\left(\begin{array}{ccc} h_{11} & h_{12} & h_{13}\\ h_{21} & h_{22} & h_{23}\\ h_{31} & h_{32} & h_{33} \end{array}\right)\left(\begin{array}{c} x_{1}\\ x_{2}\\ x_{3} \end{array}\right)$$

Or *X′* = *HX* Let the inhomogeneous coordinates *X* and *X′* in plane *A* and *A'* be (*x, y*) and (*x', y'*), respectively. The projective transformation can be written as:

(2) $$\begin{array}{l} x^{\prime }=\frac{h_{11}x+h_{12}y+h_{13}}{h_{31}x+h_{32}y+h_{33}}\\ y^{\prime }=\frac{h_{21}x+h_{22}y+h_{23}}{h_{31}x+h_{32}y+h_{33}} \end{array}$$

Rearranging Equation (2) we have:

(3) $$\begin{array}{l} h_{11}x+h_{12}y+h_{13}-x^{\prime }h_{31}x-x^{\prime }h_{32}y-x^{\prime }h_{33}=0\\ h_{21}x+h_{22}y+h_{23}-y^{\prime }h_{31}x-y^{\prime }h_{32}y-y^{\prime }h_{33}=0 \end{array}$$

Equation (3) is an over-determined system, the Direct Linear Transformation (DLT) algorithm [13] is utilized for solving the system. Rewriting Equation (3), the linear system can be expressed in matrix form:

(4) $$\text{A}Ĥ=\hat{\text{x}}$$

where $\hat{\text{x}}={\left[\begin{array}{ccccccc} x_{1}^{\prime } & y_{1}^{\prime } & x_{2}^{\prime } & y_{2}^{\prime } & \cdots & x_{i}^{\prime } & y_{i}^{\prime } \end{array}\right]}^{T}$, *Ĥ* = [*h*_11_ *h*_12_ ⋯ *h*_33_]^*T*^, and: align

(5) $$\text{A}=\left[\begin{array}{ccccccccc} x_{1} & y_{1} & 1 & 0 & 0 & 0 & -x_{1}^{\prime }x_{1} & -x_{1}^{\prime }y_{1} & -x_{1}^{\prime }\\ 0 & 0 & 0 & x_{1} & y_{1} & 1 & -y_{1}^{\prime }x_{1} & -y_{1}^{\prime }y_{1} & -y_{1}^{\prime }\\ x_{2} & y_{2} & 1 & 0 & 0 & 0 & -x_{2}^{\prime }x_{2} & -x_{2}^{\prime }y_{2} & -{x^{\prime }}_{2}\\ 0 & 0 & 0 & x_{2} & y_{2} & 1 & -y_{2}^{\prime }x_{2} & -y_{2}^{\prime }y_{2} & -y_{2}^{\prime }\\ \vdots & \vdots & \vdots & \vdots & \vdots & \vdots & \vdots & \vdots & \vdots \\ x_{i} & y_{i} & 1 & 0 & 0 & 0 & -x_{i}^{\prime }x_{i} & -x_{i}^{\prime }y_{i} & -x_{i}^{\prime }\\ 0 & 0 & 0 & x_{i} & y_{i} & 1 & -y_{i}^{\prime }x_{i} & -y_{i}^{\prime }y_{i} & -y_{i}^{\prime } \end{array}\right]$$

The algorithm is expressed as follows:

*Step 1*: For each corresponding point compute the matrix A. (I ≧ 4).
*Step 2*: Obtain the SVD (Singular value decomposition) of A.
*Step 3*: Let **A = UDV^T^**. The last column of matrix **V** corresponding to the smallest singular value of **A** is composed of the elements of the vector *Ĥ*.

### 3.4. Module Image Recognition

**Figure 12.**
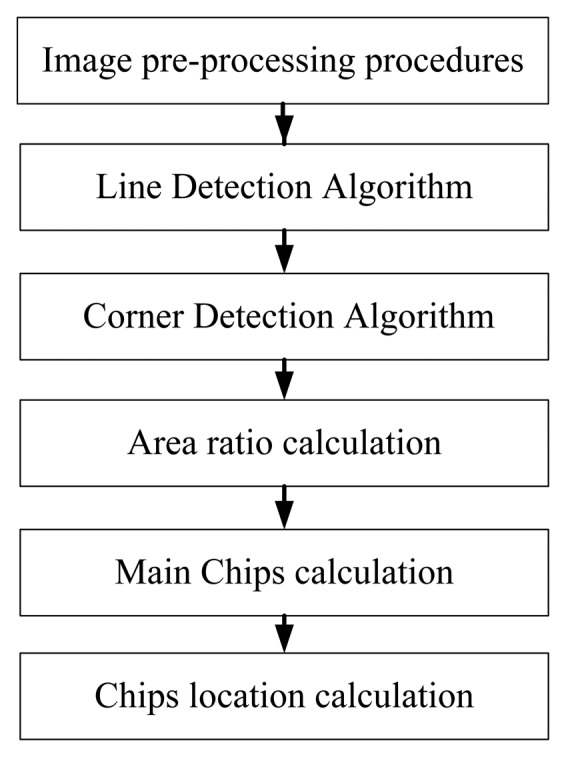
Module image recognition process.

**Figure 13.**
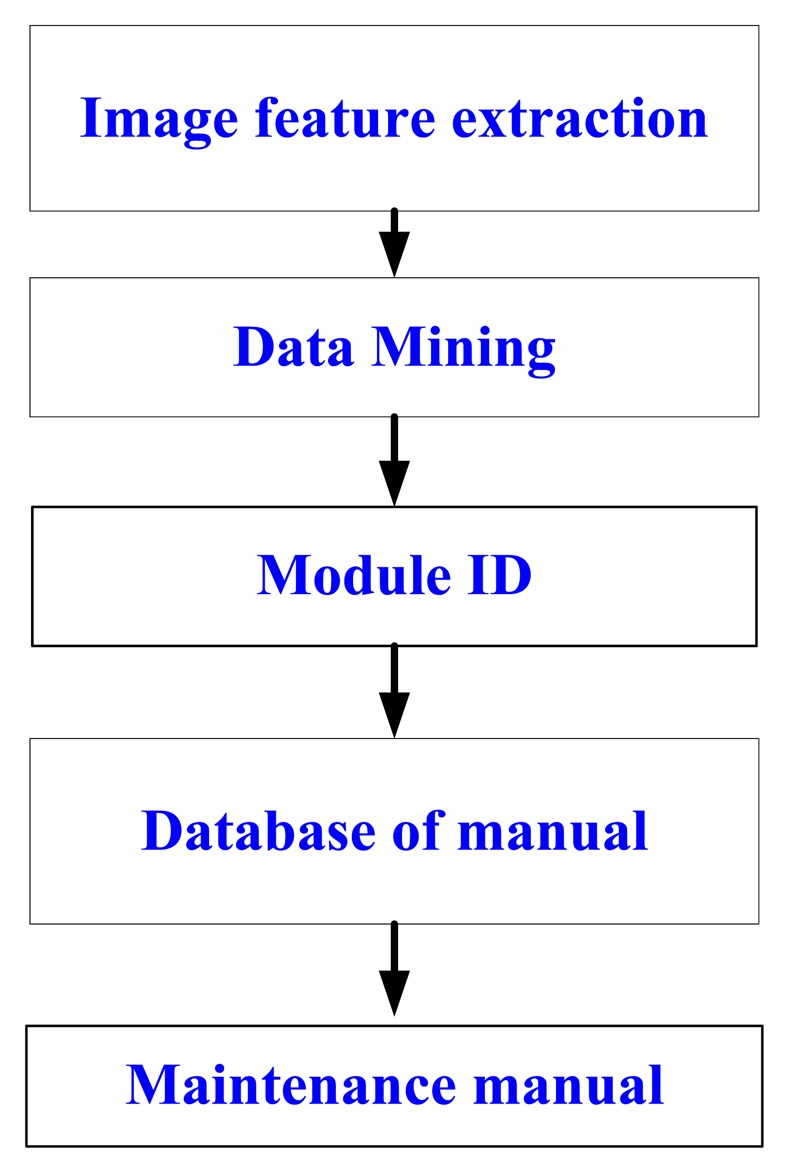
The database searching steps.

The main features of a circuit board are the shape and the number of chips. Figure 12 shows the module image recognition process. Image pre-processing procedures contain the color model transformation and the binarization. Moreover, YcbCr is chosen as the color model, which is less susceptible to the impact of the light. Then we use the Hough Transform [14] to conduct line detection and calculate the edge of the main chip module. We also use the Harris Corner Detection Algorithm [15,16] to calculate the module and the main chip corner. The main principle of Harris corner detector is to use the Gaussian filter to detect the corner response of each pixel in the image. The Gaussian filter can inhibit noise and reduce the probability of misjudgment, so the Harris corner detector has good performance for our application. The first feature of module is to calculate the area ratio of the module and main chips. The second is to calculate the amount of the main chips. The third feature is to calculate the position of the main chip in the module. The database searching steps are shown in Figure 13.

In Figure 13, the Adaptive Network-Based Fuzzy Inference System (ANFIS) is used for data mining in our database. There are five layers in ANFIS architecture [17,18] which are described as follows:

*Layer 1*: Every node i in this layer is an adaptive node with a node function *μ_j_*(*x_i_*). In this paper, the generalized Gaussian membership function: align
  (6) $$\mu_{j}(x_{i})=\exp\left[-{\left(\frac{x_{i}-c_{ji}}{a_{ji}}\right)}^{2}\right],\text{for}\;ji=\;1,\;\ldots ,k$$
  is used, where *x_i_* is the input of node *i* and *μ_j_* is a linguistic label associated with this node, and *c_ji_* , *a_ji_* are called premise parameters.
*Layer 2*: Every node in this layer is a fixed node whose output is the product of all the incoming signals:
  (7) $$w_{p}=\prod_{i=1}^{N}\mu_{ji}(x_{i}),\;\text{for}\;p=1,\ldots ,P;i=1,\ldots ,N$$
*Layer 3*: Here, the *i*th node calculates the ratio of *i*th rule's firing strength to the sum of firing strengths of all rules:
  (8) $${\bar{w}}_{p}=\frac{w_{p}}{\overset{P}{\underset{p=1}{\text{∑}}}w_{p}}$$
*Layer 4*: Every node *i* in this layer is an adaptive node with a node function:
  (9) $${\bar{w}}_{p}f_{p}={\bar{w}}_{p}\left(\sum_{i=0}^{N}r_{pi}x_{i}\right),\;x_{0}=1$$
  where *w̄_p_* is a normalized firing strength from layer 3 and *r_pi_,x_i_* are referred to as consequent parameters.
*Layer 5*: The single node in this layer is called the output node, which computes the output as the weighting average of all incoming signals:
  (10) $$\sum_{p=1}^{P}{\bar{w}}_{p}f_{p}=\sum_{p=1}^{P}w_{p}f_{p}/\sum_{p=1}^{P}w_{p}$$

**Figure 14.**
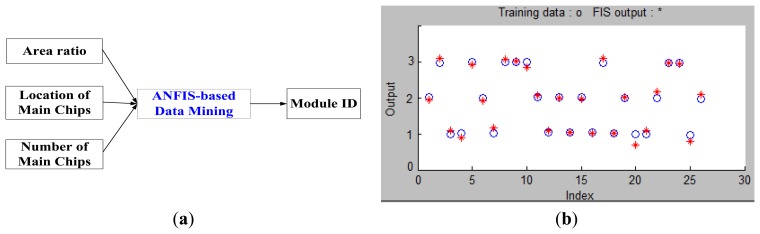
(**a**) The architecture of ANFIS-Based training system; (**b**) The ANFIS training results.

The architecture of the ANFIS-based training system is illustrated as Figure 14a. The inputs of ANFIS are the ratio of the main chip to the module, the size and position of the main chip in the module. The output is module ID. For illustration purpose, the training results of the first three sampled modules are depicted in Figure 14b in which twenty six training data marked in blue circles for each sample were used and the training results were marked with a red star. The training results suggest that the ANFIS-based system is quite effective in this application.

## 4. Experimental Results

**Figure 15.**
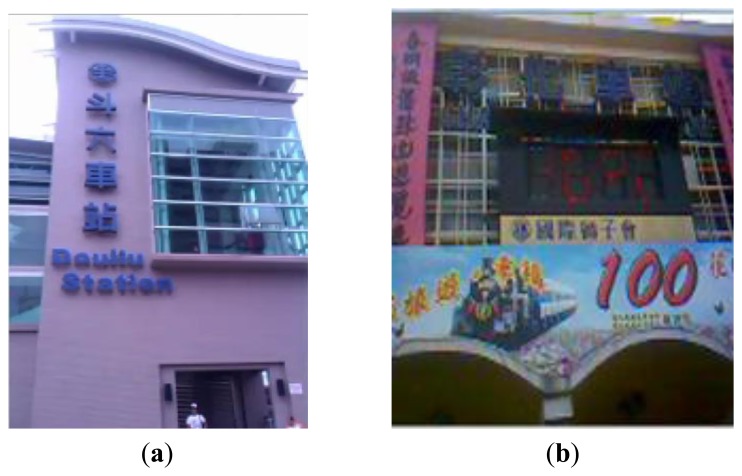
(**a**) Real-time image of the Douliou Station; (**b**) Real-time image of the Changhua Station.

The image transmission results are shown in Figure 15. The transmission distances of Figure 15a and Figure 15b are 6 km and 64 km, respectively.

**Figure 16.**
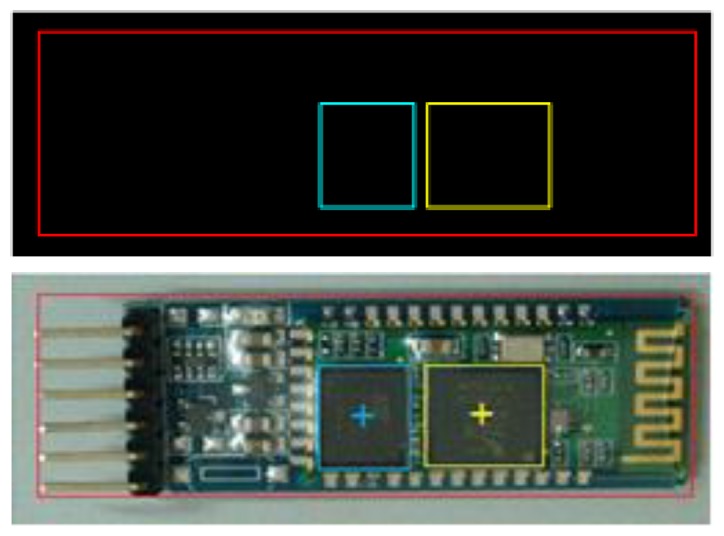
Case of Bluetooth module identification.

Figure 16 shows the module identification case. The test module is marked by the red block. Yellow and blue blocks are the identified main chips and are marked chip 1 and chip 2, respectively. The area ratio of main chip to module can be calculated from Figure 16. We use a cross to mark the center of area for the corresponding chip. In our experiment, fifteen different modules including the one in Figure 16 were taken as samples for the performance test. Though these samples are similar in appearance, they all have different circuit functionalities. The success rate for each sample is computed as follows:

$$\text{Success Rate}=\frac{\text{Number of successful identifications}}{\text{Total number of tests}}\times 100\%$$

Table 2 shows the results of QR-code and image recognition. The average recognition rate of image is 73.23%. The average computing time is 3.185 seconds. The average recognition rate of QR-code is 96.287%. The average computing time is 0.043 seconds. In practice, the threshold for successful identification rate including image recognition and QR-code recognition is set at 70%. If the success rate is below the threshold, like in the image recognition rates depicted in Table 2 for Module 14 and Module 15, respectively, they should be labelled “Failure” and sent back to the maintenance support centre for repair, as indicated in Figure 2. From Table 2, we know that QR-code recognition is better than others. After the circuit board is identified, an e-mail will be sent to repairmen. The repairmen only need to download and open the attached files. Then, the invalid modules can be repaired following the instructions.

## 5. Conclusions

In this paper, we have proposed a Logistical Remote Association Repair Framework (LRARF) to help repairmen with the maintenance operations of large-scale network systems keeping the system at a high quality of services level. The repairman can use any kind of smart mobile phone to capture QR-codes and images of fault circuit boards so the invalid modules can be recognized via the proposed algorithm. Specifically, DBMS will automatically search for the maintenance manual for the corresponding invalid modules, and transmit the maintenance instructions back to the repairman. The experimental results not only validate the effectiveness of our proposed Android-based platforms in application to the recognition of invalid modules, but also show that the live video can be recorded on the MSC synchronously.

## Figures and Tables

**Table 1.** Four-stage operations and situational modes.

**Stage**	**Situational Setting**	**Process**
1	1. Fuse is burned	Repair directly
	2. Module is identifiable	
2	1. Module is faulty	Captures the QR-Code of module and search by DBMS
	2. Module is unidentifiable	
	3. QR-Code is identifiable	
3	1. Module is faulty	Captures the image of module and search by DBMS
	2. Module is unidentifiable	
	3. QR-Code is unidentifiable neither	
4	1. Module is faulty	Service by maintenance support center
	2. Module is unidentifiable	
	3. QR-Code is unidentifiable	
	4. Image of module is unidentifiable	

**Table 2.** The results of QR-code and image recognition.

**No.**	**Image Recognition Rate (%)**	**Image Computing Time (s)**	**QR-code Recognition Rate (%)**	**QR-code Computing Time (s)**
1	66.42	3.33	98.85	0.03
2	64.84	3.91	99.51	0.01
3	76.67	1.83	97.56	0.04
4	72.36	4.32	96.36	0.08
5	81.46	3.57	97.81	0.07
6	84.01	2.69	91.78	0.04
7	70.98	4.33	99.54	0.06
8	91.03	3.12	94.44	0.08
9	78.98	1.91	93.87	0.02
10	65.77	2.31	96.78	0.06
11	54.45	3.12	96.42	0.03
12	87.65	3.44	99.32	0.01
13	85.41	4.35	99.21	0.07
14	64.21	1.23	90.32	0.03
15	54.32	4.32	92.54	0.02
